# Some Inequalities for the *q*-Analogue of the Classical Riemann Zeta Functions and the *q*-Polygamma Functions

**DOI:** 10.1155/2014/543593

**Published:** 2014-01-22

**Authors:** Banyat Sroysang

**Affiliations:** Department of Mathematics and Statistics, Faculty of Science and Technology, Thammasat University, Pathumthani 12121, Thailand

## Abstract

We present the generalizations on some inequalities for the *q*-analogue of the classical Riemann zeta functions and the *q*-polygamma functions.

## 1. Introduction and Preliminaries

Let *c* ∈ *ℂ* and *q* ∈ (0,1). The *q*-shifted factorial [1–5] is defined by

(1) $$\begin{array}{c} {\left(c;q\right)}_{\infty }=\overset{\infty }{\underset{k=0}{\prod }}\left(1-cq^{k}\right). \end{array}$$

The *q*-Jackson integral [6] from 0 to *c* is defined by

(2) $$\begin{array}{c} \overset{c}{\underset{0}{\int }}f\left(x\right)d_{q}x=\left(1-q\right)c\overset{\infty }{\underset{n=0}{\sum }}f\left(cq^{n}\right)q^{n}. \end{array}$$

The *q*-gamma function [6] is defined by

(3) $$\begin{array}{c} \Gamma_{q}\left(x\right)=\frac{{\left(q;q\right)}_{\infty }}{{\left(q^{x};q\right)}_{\infty }}{\left(1-q\right)}^{1-x},\;x\neq 0,-1,-2,\ldots . \end{array}$$

The *q*-digamma [7–9] function is defined by

(4) $$\begin{array}{c} \psi_{q}\left(x\right)=\frac{\Gamma_{q}^{\prime }\left(x\right)}{\Gamma_{q}\left(x\right)} \end{array}$$

and may be represented [10] as

(5) $$\begin{array}{c} \psi_{q}\left(x\right)=-\log\left(1-q\right)+\frac{\log q}{1-q}\overset{q}{\underset{0}{\int }}\frac{t^{x-1}}{1-t}d_{q}t, \end{array}$$

where *x* > 0.

The *q*-polygamma function [7] is defined by

(6) $$\begin{array}{c} \psi_{q,n}\left(x\right)=\psi_{q}^{\left(n\right)}\left(x\right) \end{array}$$

and may be represented as

(7) $$\begin{array}{c} \psi_{q,n}\left(x\right)=\frac{\log q}{1-q}\overset{q}{\underset{0}{\int }}\frac{{\left(\log u\right)}^{n}u^{x-1}}{1-u}d_{q}u, \end{array}$$

where *x* > 0 and *n* ∈ *ℕ*.

For any *x* > 0, we denote

(8) $$\begin{array}{c} \alpha \left(x\right)=\frac{\log x}{\log q}-E\left(\frac{\log x}{\log q}\right),\\ {\left[x\right]}_{q}=\frac{1-q^{x}}{1-q},\\ {\left\{x\right\}}_{q}=\frac{{\left[x\right]}_{q}}{q^{x+\alpha ({\left[x\right]}_{q})}}, \end{array}$$

where *E*(log⁡*x*/log⁡*q*) is the integer part of log⁡*x*/log⁡*q*.

The *q*-zeta function [11] is defined by

(9) $$\begin{array}{c} \zeta_{q}\left(s\right)=\overset{\infty }{\underset{n=1}{\sum }}\frac{1}{{\left\{n\right\}}_{q}^{s}}, \end{array}$$

where *Re*(*s*) > 1. Moreover, in [11], the *q*-analogue of the classical Riemann Zeta function is

(10) $$\begin{array}{c} \zeta_{q}\left(s\right)=\frac{1}{{\tilde{\Gamma }}_{q}\left(s\right)}\overset{\infty }{\underset{0}{\int }}t^{s-1}Z_{q}\left(t\right)d_{q}t, \end{array}$$

where *Re*(*s*) > 1 and for any *t* > 0,

(11) Zq(t)=∑n=1∞1((q−1){n}qt;q)∞,Γ~q(s)=(1+(1−q)−1)((q−1)qt;q)∞((q−1)−1q1−t;q)∞((1−q)−s(q−1);q)∞((q−1)−1;q)∞Γq(s).

In 2009, Brahim [12] proved the results as follows:

(12) $$\begin{array}{c} \psi_{q,m}\left(x\right)\psi_{q,n}\left(x\right)\geq \psi_{q,\left(m+n\right)/2}^{2}\left(x\right), \end{array}$$

where *x* > 0 and *m*, *n*, (*m* + *n*)/2 ∈ *ℕ*. Consider

(13) $$\begin{array}{c} {\left[s+1\right]}_{q}\frac{\zeta_{q}\left(s\right)}{\zeta_{q}\left(s+1\right)}\geq q{\left[s\right]}_{q}\frac{\zeta_{q}\left(s+1\right)}{\zeta_{q}\left(s+2\right)}, \end{array}$$

where *s* > 1.

In 2010, Krasniqi et al. [13] gave an inequality as follows:

(14) $$\begin{array}{c} \frac{{\tilde{\Gamma }}_{q}\left(x/a+y/b\right)}{{\tilde{\Gamma }}_{q}^{1/a}\left(x\right){\tilde{\Gamma }}_{q}^{1/b}\left(y\right)}\leq \frac{\zeta_{q}^{1/a}\left(x\right)\zeta_{q}^{1/b}\left(y\right)}{\zeta_{q}\left(x/a+y/b\right)}, \end{array}$$

where *x*, *y*, *a* > 1 and *x*/*a* + *y*/*b* > 1 = 1/*a* + 1/*b*.

In 2012, Sulaiman [10] gave the inequalities as follows:

(15) $$\begin{array}{c} \psi_{q,m/a+n/b}\left(\frac{x}{a}+\frac{y}{b}\right)\leq \psi_{q,m}^{1/a}\left(x\right)\psi_{q,n}^{1/b}\left(y\right), \end{array}$$

where *x*, *y* > 0, *a* > 1, 1/*a* + 1/*b* = 1, and *m*, *n*, *x*/*a* + *y*/*b* ∈ *ℕ*.

(16) $$\begin{array}{c} {\left(\psi_{q,m}(x)+\psi_{q,n}(y)\right)}^{1/p}\leq \psi_{q,m}^{1/p}\left(x\right)+\psi_{q,n}^{1/p}\left(y\right), \end{array}$$

where *x*, *y* > 0, *p* ≥ 1, and *m*, *n* ∈ *ℕ*.

In this paper, we present the generalizations on the above inequalities.

## 2. Results

**Theorem 1**

Let *n*_1_, *n*_2_,…, *n*_*k*_ ∈ *ℕ*, *x*_1_, *x*_2_,…, *x*_*k*_ > 0, and *p*_1_, *p*_2_,…, *p*_*k*_ > 1 be such that ∑_*i*=1_^*k*^1/*p*_*i*_ = 1 and *M* = ∑_*i*=1_^*k*^ *n*_*i*_/*p*_*i*_ ∈ *ℕ*. Then

(17) $$\begin{array}{c} \psi_{q,M}\left(\overset{k}{\underset{i=1}{\sum }}\frac{x_{i}}{p_{i}}\right)\leq \overset{k}{\underset{i=1}{\prod }}\psi_{q,n_{i}}^{1/p_{i}}\left(x_{i}\right). \end{array}$$

**Proof**

By the generalized Hölder inequality,

(18) ψq,M(∑i=1kxipi)=log⁡q1−q∫0q(log⁡u)Mu∑i=1kxi/pi−11−udqu=log⁡q1−q∫0q(log⁡u)∑i=1kni/piu∑i=1k(xi−1)/pi(1−u)∑i=1k(1/pi)dqu=log⁡q1−q∫0q∏i=1k((log⁡u)ni/piu(xi−1)/pi(1−u)1/pi)dqu≤log⁡q1−q∏i=1k(∫0q(log⁡u)niuxi−11−udqu)1/pi=∏i=1kψq,ni1/pi(xi).

One can easily check that if we put *k* = 2 in Theorem 1 then we get the following.

**Corollary 2 (see [10])**

Let *n*_1_, *n*_2_ ∈ *ℕ*, *x*_1_, *x*_2_ > 0, and *p*_1_, *p*_2_ > 1 be such that 1/*p*_1_ + 1/*p*_2_ = 1 and *n*_1_/*p*_1_ + *n*_2_/*p*_2_ ∈ *ℕ*. Then

(19) $$\begin{array}{c} \psi_{q,n_{1}/p_{1}+n_{2}/p_{2}s}\left(\frac{x_{1}}{p_{1}}+\frac{x_{2}}{p_{2}}\right)\leq \psi_{q,n_{1}}^{1/p_{1}}\left(x_{1}\right)\psi_{q,n_{2}}^{1/p_{2}}\left(x_{2}\right). \end{array}$$

It is easy to notice that if we put *k* = *p*_1_ = *p*_2_ = 2 and *x*_1_ = *x*_2_ = *x* in Theorem 1 then we get the following.

**Corollary 3 (see [12])**

Let *n*_1_, *n*_2_ ∈ *ℕ* and *x* > 0 be such that (*n*_1_ + *n*_2_)/2 ∈ *ℕ*. Then

(20) $$\begin{array}{c} \psi_{q,\left(n_{1}+n_{2}\right)/2}^{2}\left(x\right)\leq \psi_{q,n_{1}}\left(x\right)\psi_{q,n_{2}}\left(x\right). \end{array}$$

**Theorem 4**

Let *n*_1_, *n*_2_,…, *n*_*k*_ ∈ *ℕ*, *x*_1_, *x*_2_,…, *x*_*k*_ > 0, and *p* ≥ 1. Then

(21) $$\begin{array}{c} {\left(\overset{k}{\underset{i=1}{\sum }}\psi_{q,n_{i}}(x_{i})\right)}^{1/p}\leq \overset{k}{\underset{i=1}{\sum }}\psi_{q,n_{i}}^{1/p}\left(x_{i}\right). \end{array}$$

**Proof**

First, we note that

(22) $$\begin{array}{c} \overset{k}{\underset{i=1}{\sum }}a_{i}^{p}\leq {\left(\overset{k}{\underset{i=1}{\sum }}a_{i}\right)}^{p} \end{array}$$

for all *a*_1_, *a*_2_,…, *a*_*k*_ > 0. Then

(23) (∑i=1kψq,ni(xi))1/p =(∑i=1k(log⁡q1−q∫0q(log⁡u)niuxi−11−udqu))1/p =(log⁡q1−q)1/p(∫0q∑i=1k((log⁡u)ni/pu(xi−1)/p(1−u)1/p)pdqu)1/p ≤(log⁡q1−q)1/p(∫0q(∑i=1k(log⁡u)ni/pu(xi−1)/p(1−u)1/p)pdqu)1/p.

By Minkowski's inequality,

(24) (∑i=1kψq,ni(xi))1/p ≤(log⁡q1−q)1/p∑i=1k(∫0q((log⁡u)ni/pu(xi−1)/p(1−u)1/p)pdqu)1/p =∑i=1k(log⁡q1−q∫0q((log⁡u)niuxi−11−u)pdqu)1/p =∑i=1kψq,ni1/p(xi).

One can easily check that if we put *k* = 2 in Theorem 4 then we get the following.

**Corollary 5 (see [10])**

Let *n*_1_, *n*_2_ ∈ *ℕ*, *x*_1_, *x*_2_ > 0, and *p* ≥ 1. Then

(25) $$\begin{array}{c} {\left(\psi_{q,n_{1}}(x_{1})+\psi_{q,n_{2}}(x_{2})\right)}^{1/p}\leq \psi_{q,n_{1}}^{1/p}\left(x_{1}\right)+\psi_{q,n_{2}}^{1/p}\left(x_{2}\right). \end{array}$$

**Theorem 6**

Let *x*_1_, *x*_2_,…, *x*_*k*_, *p*_1_, *p*_2_,…, *p*_*k*_ > 1 be such that ∑_*i*=1_^*k*^ *x*_*i*_/*p*_*i*_ > 1 = ∑_*i*=1_^*k*^1/*p*_*i*_. Then

(26) $$\begin{array}{c} \zeta_{q}\left(\overset{k}{\underset{i=1}{\sum }}\frac{x_{i}}{p_{i}}\right)\leq \frac{\prod_{i=1}^{k}{\tilde{\Gamma }}_{q}^{1/p_{i}}\left(x_{i}\right)}{{\tilde{\Gamma }}_{q}\left(\sum_{i=1}^{k}x_{i}/p_{i}\right)}\overset{k}{\underset{i=1}{\prod }}\zeta_{q}^{1/p_{i}}\left(x_{i}\right). \end{array}$$

**Proof**

By the generalized Hölder inequality,

(27) ζq(∑i=1kxipi) =1Γ~q(∑i=1kxi/pi)∫0∞t∑i=1kxi/pi−1Zq(t)dqt =1Γ~q(∑i=1kxi/pi)∫0∞t∑i=1k(xi−1)/piZq∑i=1k1/pi(t)dqt =1Γ~q(∑i=1kxi/pi)∫0∞∏i=1k(txi−1Zq(t))1/pidqt ≤1Γ~q(∑i=1kxi/pi)∏i=1k(∫0∞txi−1Zq(t)dqt)1/pi =1Γ~q(∑i=1kxi/pi)  ×∏i=1k(Γ~q1/pi(xi)(1Γ~q(xi)∫0∞txi−1Zq(t)dqt)1/pi) =∏i=1kΓ~q1/pi(xi)Γ~q(∑i=1kxi/pi)∏i=1kζq1/pi(xi).

It is easy to notice that if we put *k* = 2 in Theorem 6 then we get the following.

**Corollary 7 (see [13])**

Let *x*_1_, *x*_2_, *p*_1_, *p*_2_ > 1 be such that *x*_1_/*p*_1_ + *x*_2_/*p*_2_ > 1 = 1/*p*_1_ + 1/*p*_2_. Then

(28) $$\begin{array}{c} \frac{{\tilde{\Gamma }}_{q}\left(x_{1}/p_{1}+x_{2}/p_{2}\right)}{{\tilde{\Gamma }}_{q}^{1/p_{1}}\left(x_{1}\right){\tilde{\Gamma }}_{q}^{1/p_{2}}\left(x_{2}\right)}\leq \frac{\zeta_{q}^{1/p_{1}}\left(x_{1}\right)\zeta_{q}^{1/p_{2}}\left(x_{2}\right)}{\zeta_{q}\left(x_{1}/p_{1}+x_{2}/p_{2}\right)}. \end{array}$$
